# Eliciting Egg Consumer Preferences for Organic Labels and Omega 3 Claims in Italy and Hungary

**DOI:** 10.3390/foods9091212

**Published:** 2020-09-01

**Authors:** Ching-Hua Yeh, Davide Menozzi, Áron Török

**Affiliations:** 1 Institute for Food and Resource Economics, University of Bonn, 53115 Bonn, Germany; chinghua.yeh@ilr.uni-bonn.de; 2 Department of Food and Drug, University of Parma, 43124 Parma, Italy; davide.menozzi@unipr.it; 3 Department of Agricultural Economics and Rural Development, Corvinus University of Budapest, 1093 Budapest, Hungary

**Keywords:** egg, production method, nutrition and health claims, choice experiment, random parameter logit model, latent class analysis

## Abstract

This paper investigates consumers’ preferences for egg purchase in two European countries, Hungary and Italy. We utilize random parameter logit models to interpret the results of discrete choice experiments (DCE) for the elicitation of preference of the egg consumers. A sample of 403 in the Hungarian survey and 404 in the Italian survey were recruited in summer 2018. The DCE questionnaire includes the following product and process characteristics: organic labels, nutrition and health claims, and price. Our results show that for Hungarian and Italian consumers, the price is the most important attribute, followed by the nutrition and health claim and the organic production labelling. Three egg consumer segments can be identified via latent class models for each country. In both countries, we found similar consumer groups, the Price Sensitive and Quality Optimizing Opportunist Consumers and Health Conscious Buyers, respectively. Particularly, compared to the other segments the Health Conscious Buyers (46% in Hungary and 49% in Italy) exhibited stronger preference for and are willing to pay a higher price premium for eggs with organic label and nutrition claims. In Italy, we identified a third segment with consumers preferring simpler labelling approach, whilst in Hungary we found a consumer segment distrusting the EU organic logo.

## 1. Introduction

### 1.1. Organic Labelled Foods in the European Union

In the European Union (EU), organic food must comply with the principles of organic production, certification and labelling of Regulation (EU) 2018/848 of the European Parliament and of the Council. Since the first European organic regulation (Council Regulation EEC 2092/91 of 24 June 1991), organic food production plays an essential role in both the EU’s food quality systems and rural development. On the community level, since July 2010 the organic logo (green leaf), introduced by Commission Regulation (EU) No 271/2010, is compulsory for all the pre-packed organic foods and voluntary for non-pre-packed foods. Besides the EU logo, in several European countries other voluntary organic certification labels are managed by different organizations, e.g., the French “AB—agriculture biologique”, or the national “Bio”—label in Germany. Though the EU organic label has been in use for almost a decade, its recognition among European consumers is not too high and significantly differs among member states. According to recent surveys by the European Commission [1,2,3,4], generally Northern and Western European citizens know the label the most (34–60%), while among Southern and Eastern European the level of recognition was quite low (9–17%).

Not only the label recognition, but also the market share of organic foods differs significantly in the EU. Based on the yearly per capita spending on organic food, Denmark, Sweden, Luxemburg and Austria are the countries where the demand significantly exceeds the EU average (EUR 50 per capita per year). Contrarily, in some Eastern and southern EU member states (e.g., Estonia, Greece, Malta and Portugal) demand for organic labelled food almost does not exist [5].

### 1.2. Egg Market and Consumption

Hen egg is consumed worldwide as a valuable and cheap source of protein. Egg producers are in possession of practices required for producing eggs with specific attributes; therefore, they are easy to be classified into distinct product categories [6]. After a setback in the early ‘90s, per capita egg consumption in Europe had achieved around 13.3 kg [7] and expected to increase to 14.3 kg per capita per year by 2030 [8].

However, in the second half of the 20th century, household egg consumption faced a significant fall in several developed countries (e.g., UK, USA) for several reasons, including changing diets and habits, animal welfare concerns, and health-related issues [9,10,11]. Additionally, an increase in egg consumption was identified among vegetarians and consumers who avoid red meat [11], and as a consequence of the income decline, given the inelastic demand [10]. Brand, in this sector, is less important than the size, the number of eggs in the pack and variety [11]. However, several studies have analyzed the market segments; based on a consumer survey, in the UK there was a polarization of egg consumers: animal-friendly consumers requiring free-range eggs at one corner and those who care more about functional properties (e.g., size and shell) and the value for money [11]. UK consumers are willing to pay a substantially higher price for eggs with animal-friendly production method than the technology change (e.g., cage-free) that actually would increase the production costs [9].

According to a survey conducted in France, buying organic eggs is not affected by socio-economic factors (e.g., income, age, family size) except for the education level. Purchasing other organic products (e.g., milk) increases the probability of buying organic eggs [12]. Spanish consumers are willing to pay a positive price premium for eggs produced organically as well as for the specification of the production origin of eggs [13]. In Norway, there is a segment of consumers willing to pay a substantial premium for organic eggs, but this segment is limited in size. Other consumers do not seem to appreciate the value added of organic eggs, presenting a decreasing marginal utility for added attributes. In other words, organic labelled eggs are competing with, e.g., free-range eggs, where less costly labelled eggs are able to gain a competitive advantage [14].

In Hungary, the highest egg consumption was realized right before the political change at the end of the 80s, and since then domestic consumption is characterized by a declining trend, due to several reasons, including the lowering purchasing power [15]. Price-sensitive Hungarian consumers seek for the fresh and sound eggs, and consider egg consumption as part of a preferable and healthy diet [16].

In Italy, the average egg consumption is in line with the EU statistics (13.4 kg per capita) and has been relatively stable over the past 20 years [17]. It is estimated that approximately 35% of this quantity is used in the food industry in the form of egg products. Italy is self-sufficient for the consumption of eggs, i.e., produces enough eggs to cover the entire national needs [18].

In general, the EU’s egg supply is enough for domestic consumption, the self-sufficiency was around 103% in recent years [8], and the level of national production has covered the level of domestic consumption both in Hungary and Italy [8]. In addition, the supply of organic egg production is highly concentrated, where the top five biggest egg producers in the EU have a market share of 76%. In recent years, Italy was the seventh biggest organic egg producer (6.9%), while the Hungarian production was almost negligible [19].

### 1.3. Consumers’ Preference and Perception of Organic Labelled Food

In the past few decades, food consumption patterns have been changing rapidly due to the increasing concerns about food quality and safety, health benefits, environmental impacts and animal welfare concerns. This leads to consumer interest in organic food which is free from pesticides and chemical residues and boosts consumer demand for differentiated agricultural products in the food markets.

Kiss et al. [20] and Gergely et al. [21] found that, in general, organic food is considered more expensive compared to conventional ones among Hungarian consumers, and this is the most significant barrier why it is not consumed widely. The premium price level was also confirmed by previous field research that the organic foods cost more and had a 56–85% price premium compared to their conventional counterparts [22]. However, families with children consume more organic foods [21] and vegetarians are more likely to become organic consumers because their primary motivation is to avoid bad quality products, though natural-like products are usually confusing whether they are organic [23]. The typical Hungarian organic food consumers are female, live in a larger city, belong to the upper-income level segment and are committed to a healthy lifestyle and environmental protection [22,24]. Hungarian’s organic consumption is highly influenced by food prices and individuals’ income levels, and their main motivations are to follow a healthy diet and to avoid diseases and other health-related reasons [25]. Based on the case of the organic milk market in Hungary, Szente et al. [26] suggested that low price category and local/regional product strategy together with prestige product strategy should be implemented in order to increase the level of organic consumption. Among organic consumers, the average willingness to pay (WTP) is 30–50%; however, a low level of recognition for such products is common [27]. Regarding the Hungarian consumers’ attitude towards organic labelled products, it has been found that they are sensitive for sustainability issues [22]. However, organic food consumption has remained relatively low due to the lack of trust in organic products, and consumers cannot distinguish them from regular ones [28]. A recent consumer survey highlighted that freshness, flavor, positive impacts on health, ingredients and to be free from additives are the most important characteristics that influence consumers when buying organic foods [24].

Italy is the fifth organic market worldwide, following the US, Germany, France and China, with more than EUR 3.1 billion retail sales and 3.2% of market share in 2017. Italy is also a net exporter of organic products, with a value of total exports higher than EUR 2 billion in 2017 [5]. In general, Italian women and young people show a higher intensity of organic food consumption and WTP for organic products [29,30]. The presence of children in the family results in a higher WTP for organic products too [30]. Familiarity is another factor influencing organic consumption and WTP, since consumers who regularly purchase organic foods are also willing to spend more for these products [30]. The organic label is more familiar among young adults, in terms of both visibility and understanding. It was suggested that this higher visibility and familiarity can be justified by the high market penetration of organic products at the national level [31].

A cross national study has shown that in Italy the EU logo was widely used, also because neither a governmental nor a dominant private organic logo existed. This factor could also explain the positive attitude and trust of Italian consumers towards the EU logo [32]. The trust in organic labels also has a positive impact on attitude towards and WTP for organic food. For this reason, it was studied that providing additional production information about organic farming practices did not affect the WTP for organic products [30]. In other words, the EU organic logo was for these consumers self-explanatory. Among the different supply chain actors, it was demonstrated that trust in farmers is a significant predictor of the intention to purchase organic products [33]. Italian organic consumers exhibit a higher level of sustainability concern in their general food choices, adhere to more sustainable consumption principles and have a more sustainable lifestyle [29,34]. Those consumers attaching a higher importance to health aspects and naturalness of food, and are concerned over food safety, exhibit a higher organic consumption intensity [29], and are willing to pay more for organic products [30].

In general, consumers usually perceive organic eggs as being healthier than conventional products, and they are willing to pay more for them [13,35,36,37]. Andersen [36] found a high and positive correlation between Danish consumers’ revealed WTP a premium for organic and free-range eggs, indicating a similar perception of organic eggs with eggs carrying labels indicating improved animal welfare. Consumers’ WTP was higher for organic, especially among wealthier, urban and more educated consumers [36]. In Denmark, consumers are ready to pay a significant price premium (58%) for organic eggs. [38]. In Spain, 80% of consumers were willing to pay a price premium for organic eggs (EUR 0.85 for a six package) [39] and organic and local claims were complements for many of the consumers [13]. For Italian occasional organic consumers, locally produced foods (including eggs) are considered to have a higher quality level than organic, as the latter was found to be more distant [40]. In the survey of Gerini, Alfnes and Schjøll [14], the Norwegian consumers were divided into three segments according to their organic purchase frequency (always/occasionally/never). They found that only regular organic buyers willing to pay more for organic eggs, while those are never buying organic foods are avoiding organic eggs even when they cost the same as the other eggs. Similar results were obtained by Heng and Peterson [41] with a US sample.

In the USA due to a Salmonella outbreak, half a billion eggs were recalled in 2010. Li et al. [42] compared results of WTP using auction experiments before and after this food safety scare. The results show that the scandal had no significant effect on the preferences for organic and conventional eggs. However, respondents that were supplied with negative information of the recall were willing to pay significantly more for organic eggs. Among Polish egg consumers, price and farming system had the most important effect on preferences, and free-range eggs were preferred more than organic ones [43]. In Germany, the organic attribute of eggs was ranked after “free of antibiotics” both in raw and processed (pasta containing egg) format: WTP estimate for the organic egg was EUR 0.23 while for pasta only EUR 0.02 [44]. Swiss consumers would pay almost 32% more for organic eggs [45]. Teixeira et al. [46] investigated emerging countries in terms of consumers’ views on egg farming and purchasing habits. In Brazil and Chile, they found that egg consumers care the most for animal welfare, and more than half of them are willing to pay at least 5% more for eggs produced with animal-friendly methods.

### 1.4. Importance of Nutrition and Health Claims for Eggs

To avoid a situation where product claims could mask the overall nutritional status of a food product and mislead consumers when purchasing food, Regulation (EC) No 1924/2006 introduced the concept of nutrient profiles. This regulation seeks to protect consumers from misleading or false claims and make it easier for producers and processors to identify nutrition and health claims that can be used on specific food products [47].

Eggs usually have many desirable attributes that might attract consumers’ attention (among others, cage-free, organic, locally produced, nutrient enhanced, additive-free, etc.). Heng and Peterson [41] tried to investigate how these attributes interact and affect the overall price premium in the US market. They identified four consumer groups and found that the consumer segment of the “attribute seekers” are in favor of all labelled attributes except the health-related (omega 3) claim. A previous cross-country study investigated that almost 40% of the EU citizens stated that they buy eggs from animal welfare production (free-range or outdoor) and less than 20% admitted not to pay attention to the type of production system [48]. A US survey found that American egg consumers think that animal-friendly production methods contribute to the higher egg quality level, and a majority of them would pay more for such products. In another US study, Heng et al. [35] reported that most respondents in their study willingly paid a price premium for eggs from organic farms, outdoor access, and cage-free housing. Additionally, consumers paid more attention to animal friendly conditions than to organic production in the choice of eggs. However, consumers’ attitude towards nutrient improved eggs (e.g., omega 3 enhanced) are not clear in terms of WTP [41]. Only health-conscious Canadian consumers are willing to pay a remarkable price premium for omega 3 enhanced eggs, and their stated WTP is lower than the actual market price difference compared to generic eggs [49].

Given the above background, this paper first aims to assess consumers’ attitude towards, interest to, and willingness to pay for differentiated eggs with respect to production methods (i.e., organic) and nutrition and health claims, in two European countries, Hungary and Italy. This work uses the stated preferences approach through hypothetical choice experiments to estimate consumer demand and can help advance the understanding of the synergies or trade-offs between organic label with other nutrition and health claims. Secondly, the current study also aims to segment the country-specific consumers’ egg purchase decisions, based on the two attributes: organic label, and nutrition and health claims. We explicitly considered consumers’ heterogeneous preferences, in the light of individuals’ choices and characteristics such as socio-demographics, food purchase habits, and consumers’ food value regarding motives of food purchase, etc., identifying different consumer segments in each country. Thirdly, the final aim of this study is to estimate consumers’ WTP for differentiated eggs at the segmentation level in order to evaluate the product’s position in the market. Although the results of our study do not claim to cover the whole complex eggs market, they are expected to help practitioners and actors in the egg industry to better understand consumer motivations when buying eggs. This might further help them to target their customer segments, to develop and implement strategies in differentiated egg markets, as well as to evaluate the potential opportunities. In sum, this study extends the understanding of the trade-offs or synergies between organic labels with other nutrition and health claims, identifying consumer segments with similar behavioural and preference patterns.

In the following sections, we describe the experimental design and producers (Section 2), analytic results (Section 3), and a discussion and conclusion (Section 4).

## 2. Materials and Methods

### 2.1. Theoretical Framework

In the present study, an egg-purchasing scenario was simulated using discrete choice experiments (DCE). DCE methodology has been frequently conducted in the last decades in the marketing field e.g., [50,51,52] as a popular method to elicit consumer preferences for a product or a service and the attributes that comprise such a product or service. A DCE simulates consumers’ purchase decision and has proven to be predictive for consumers’ behavior on the market [53]. Participants are usually confronted with different sets of the choice situation and requested to indicate which of the several egg alternatives they would like to choose or to purchase. When making their choice or purchase decision, participants are forced to make a trade-off between different attributes and levels, thereby revealing their preferences.

The DCE is analyzed within a random utility framework [54,55], which, in our case, assumes that preference for eggs is a function of the utility or value of that egg’s individual attributes plus an error term and allows the analysis of the stated choice under utility maximization. For each individual i, the utility function of an alternative j is a function of the product and process characteristics $x_{ijt}$ in the choice set t. Each individual i maximises his/her utility when choosing between J alternatives can be specified as follows:

(1) $$U_{ijt}=\beta_{i}x_{ijt}+\varepsilon_{ijt}$$

where $U_{ijt}$ is the utility that individual i obtains from alternative j at the situation t; $\beta_{i}$ is a vector of parameters of variables for individual i representing his/her preferences; $x_{ijt}$ is a vector of observed attributes, and $\varepsilon_{ijt}$ is the i.i.d. extreme value type 1 stochastic error term. Principally, the standard analytic practice is to pool DCE choice data from individuals and estimate an aggregated model applying multinomial logistic regression. Because participants are assumed to have heterogeneous preferences and differ in error variances, it is important to consider these individual preferences and heterogeneity in the modelling process. To date, there has been number of modelling attempts to allow for eliciting heterogeneous preferences in DCE analysis. The state-of-the-art practice is the random parameters logit modelling (RPL) approach [56], which extends the traditional multinomial logit models by allowing parameters to randomly vary across individuals. This is computed by involving a respondent-specific stochastic component that specifies the individual specific deviation from the overall utility mean [56,57]. Following Train [56] and Hensher and Johnson [58], the probability of individual i choosing alternative j is an integral of standard logit probabilities over the parameter densities, which can be denoted as:

(2) $$P_{itj}\;\left(\theta \right)=\int \frac{\exp\left(\beta_{i}x_{ijt}\right)}{\sum \exp\left(\beta_{i}x_{ijt}\right)}f(\beta_{i}|\theta )d\beta_{i}$$

where θ is the parameter vector that specifies the distribution of β across sampling participants. In the present study, we applied the application of the RPL models using hierarchical Bayesian estimation on the effect-coded choice data. This hierarchical Bayesian choice modelling approach was motivated by the fact that compared to the classical maximum likelihood RPL models, the hierarchical Bayesian approach is known for its higher accuracy when heterogeneity in preference of the investigated population increases [59]. The hierarchical Bayesian RPL model involves two hierarchical steps [56,60,61]: in the first hierarchical step, the individual-level parameters are computed via a multivariate normal distribution characterized by a vector of mean values and a matrix of covariances. In the second hierarchical step, given an individual-level parameter, participant’s likelihood of selecting specific products/alternatives can be further estimated by an aggregated logit model.

In addition, another alternative modelling strategy to discover the heterogeneous preference is Latent Class Analysis (LCA) [56]. In the present study, we follow the LCA procedure implemented by Boxall and Adamowicz [61], which is also called as finite-mixture model, where the finite mixture accommodates interpersonal heterogeneity in preference for the observed attributes describing choice alternatives LCA assumes that the overall preference distribution is constituted by a combination of unobservable latent segments that heterogeneous in their utility between the segments but are homogeneous within the segment [56,62,63]. For the LCA, the individuals are assumed to belong to a class s with a certain probability $C_{is}$ for s=1,…,S (where $C_{is}>0$ and $\sum C_{is}=1$; S denotes the total number of classes). Thus, the probability of an individual’s membership of segment *s* will take the following form:

(3) $$C_{is}=\frac{\exp\left(\alpha \lambda_{S}\right)}{\sum_{s=1}^{S}\exp\left(\alpha \lambda_{S}\right)}$$

where $\lambda_{S}$ denotes a vector of the segment specific parameters and α is the scale factor that is assumed to be equal to one, so each participant has a probability of belonging to a particular segment [61]. For conducting LCA, individual i′s choice probability for alternative j in choice situation t can then be given as [53,56,58]:

(4) $$P_{ijt}=\overset{S}{\underset{s=1}{\sum }}C_{is}\frac{\exp\left(\beta_{s}x_{ijt}\right)}{\sum_{j=1}^{J}\beta_{s}x_{ijt}}$$

The present paper conducted classical LCA using the maximum likelihood approach. Besides estimating preferences for different consumer classes, the LCA models also provide the probability of each class membership for each individual. This is modelled as a function that participant i is classified into class s under the assumption that the stochastic error terms in membership likelihood function are i.i.d. across individuals and classes. We need to note that the LCA does not assign each individual to a particular class, but assigns to each individual a probability of membership in every class [56]. Thus, due to the properties of the RPL and LCA methods, the RPL and LCA were selected for the analysis of the country-specific DCE data in this paper to simultaneously approximate part-worth utility parameters and class membership from the DCE choices.

Moreover, we further compute country-specific segment-specific WTP estimates for each attribute level for the identified consumer segments by dividing the respective attribute level coefficient by the price coefficient. Because of involving the effect coding process in the discrete choice model, we calculated the mean WTP according to the following specification reported in Bech and Gyrd-Hansen [64]:

(5) $$\;WTP=\left(\frac{-2\;\beta_{attrtibute\;level}}{\beta_{price}}\right)$$

Finally, we utilized the Kruskal–Wallis non-parametric test [65] to further examine whether different (country-specific) consumer segments significantly differs with respect to the demographic information, purchase behaviour and food value. Kruskal–Wallis test is widely used to compare three or more independent groups that are the same or different on respective variables of interest. When the statistics of the Kruskal–Wallis statistic is calculated as statistically significant, it indicates that at least one of the compared groups is significantly different from the others.

### 2.2. Survey Instrument and Design

The selection of eggs as the studying object in this study was based on the fact that eggs are usually used as a food ingredient, represent an important part of the human diet, and are highly differentiated by the product and process attribute, e.g., nutrition and health claims. The nutrition claims allowed for regular eggs are regulated in the EU by the Regulation (EC) n. 1924/2006 of the European Parliament and of the Council. Given the role that consumers play in the decision-making process of egg purchase, it is important to strengthen our understanding of their preference for, and respond to, different information to the labelling of egg products. The online cross-country questionnaire with respect to egg purchase was devised together with academic researchers specializing in the field of agricultural and nutrition science, and consisted of three parts.

The questionnaire started with a welcome address and statement of research motivation. The first part of the questionnaire included screening questions whether the participant was (partly) responsible for their household food purchase, currently lives in the respective country, and consumed egg in the last three months. The DCE questions constituted the second part of the survey. For the choice experiment, the appropriate eggs’ attributes and their respective levels as well as an adequate egg-purchasing scenario were designed and defined. By conducting an internal discussion among academic researchers and market experts, we defined egg’s attributes that were repeatedly discussed as influencing the purchase decision of the egg on the market, were proved influential in previous studies (e.g., Carlsson et al. [66], Heng et al. [67]), and relatively independent of one another and could be modified to impact consumers’ decision making. Three attributes were included in the present cross-country study (namely, organic labels, nutrition and health claims, and price), which vary by defined levels in order to describe the choice situations. Table 1 presents the attributes and the respective levels used in the cross-country DCE setting.

**Figure 1:**
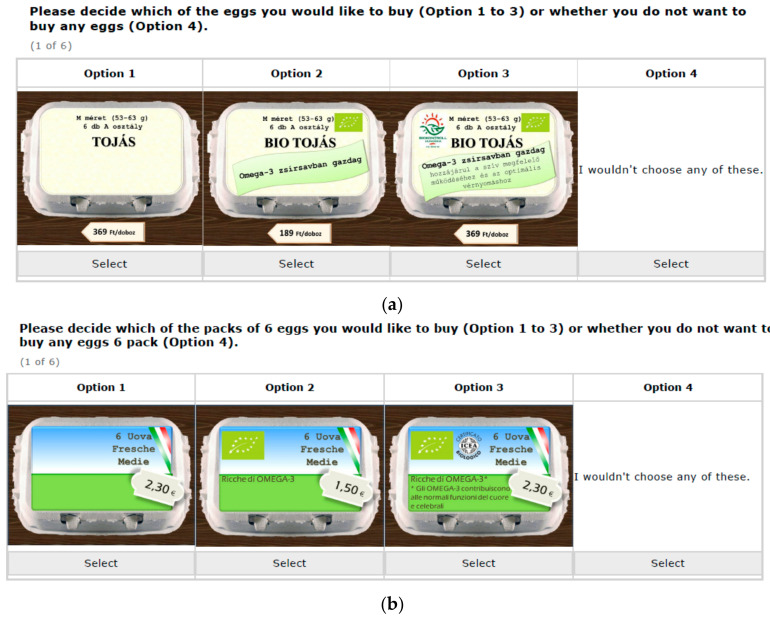
(**a**) An example of Hungarian discrete choice experiment (DCE) question. (**b**). An example of Italian DCE question.

In the introduction to the DCE question, participants were explained briefly about the upcoming purchase task using the “cheap talk script” [68,69]. For the operation of DCE questions, participants were asked to make a purchase decision among the three presented egg products and an opt-out option. The inclusion of an opt-out option in the DCE has been considered to improve the realism of the DCE. Based on an artificially created image of egg products, the combination of attributes and levels of the DCEs were graphically represented. Figure 1a,b provide examples of the DCE choice situation used in the Hungarian and Italian online surveys, respectively. The DCE applied in the present study has an unlabelled design, with three alternatives and an opt-out egg alternative. Due to a large number of total possible choice sets (${\left(3\times 3\times 4\right)}^{3}=46,656$), a reduced D-optimal design based on three egg product alternatives was employed using Ngene software (Version 1.2, ChoiceMetrics, Sydney, Australia) [70] to construct the DCE design plan. The D-optimal design seeks to maximize the logarithm of the determinant of the information matrix and consequently minimizes the determinant of the covariance matrix of the parameter estimates. In total, 20 blocking versions of the DCE questionnaire were generated in order to limit the context effect and response fatigue. Each version of the DCE survey was randomly assigned to each participant and each participant was requested to answer six DCE purchasing questions.

The third part of the questionnaire contained Likert scaling questions about participants’ perception and purchase habits with respect to egg shopping, as well as the food value questions regarding the importance of motives underlying the food choice. In the fourth part of the questionnaire, we also include items measuring demographic information and socioeconomic status. After completion of the online questionnaire, the participants were thanked for their participation.

The cross-country questionnaire was firstly developed in English in close collaboration between Hungarian and Italian researchers, and subsequently translated to the local language. Back-translations were carefully examined and led to a minor modification of the questionnaire. Finally, the online questionnaire was then pretested in order to ensure that no further changes appeared necessary in the survey.

### 2.3. Data Collection

A random and nationwide sample was drawn from population defined as adult shoppers above 18 years of age, living in the studied country (Hungary and Italy), at least partly responsible for their household food shopping, and have bought eggs in the last three months. Data were collected via cross-country online surveys in summer 2018 via the marketing research institute, LiGHTSPEED (http://www.lightspeedresearch.com/). The average completion time was about 10 min.

## 3. Results

In total, 807 usable questionnaires were obtained and deemed valid for further analysis. Table 2 shows the demographics statistics of the cross-country sample. In general, participants in both countries are half female, mostly well-educated, and living in the urban area. According to our descriptive outputs, the mean age of our participants was higher than the national statistics. Moreover, we notice that the share of female Hungarian respondents was slightly lower than the national average. Overall, the differences of the sample structure between Hungarian and Italian data are not substantial except that, in both countries, participants consisted of more post graduated respondents living in urban areas and with less children in their households. These sample characteristics should be noted when interpreting the estimated results of the DCE data, considering all the well-known limitations of online surveys highlighted in the literature (e.g., [71]).

### 3.1. RPL Model Estimates

In the first step of the DCE analysis, a country-specific random parameter logit model (RPL) was estimated for the Hungarian and Italian DCE data, assuming that there is consumer preference heterogeneity. Table 3 presents the results for the attribute importance measures of the cross-country DCE choice data. Our results reveal that in both countries price is the most important attribute, followed by the information of nutrition and health claims with respect to omega 3, while the organic labels are seen as the least important.

Table 4 shows the results for the country-specific RPL models. For each random parameter, the estimated average utility mean value and standard deviation are reported using zero-centred measures. They represent the relative attractiveness of the levels within each attribute in the numerical format expressing that the higher the number the more preferable it is to participants and conversely, the lower (more negative) the number, the less preferable it is to participants. The average utility of the opt-out alternative is computed as the mean value of the individual specific constants, and as expected, the constants in the bottom row of Table 4 are all negative and significant. This implies that consumers in both countries generally prefer selecting one of the egg alternatives in the DCE tasks. Our RPL results in both countries also show that participants are in favor of organic eggs in relation to the conventional ones, and prefer the eggs promoted by information of nutrition and health claims compared to the ones that have no nutrition and health claims. Particularly, in both countries we also found a stronger preference of eggs associated with a detailed (full) information of nutrition and health claims compared to eggs that carry only a simplified (short) nutrition claim. More specifically, this preference is considerably stronger for Italian consumers compared to Hungarian consumers. In addition, as expected our RPL outputs also reveal that participants’ preference utility decreases with an increase in price which is in line with a negative price elasticity of demand. Finally, the estimated standard deviation of the random coefficients, i.e., “No label” in Hungarian data, shown in Table 4 is 32.54 indicating heterogeneity across participants in the population with respect to the effect of “No label” information. Additionally, our results also find that the standard deviation of the random parameters are statistically significant across countries, which implies that there is a substantial heterogeneity in preference regarding the respective characteristics when purchasing eggs across consumers, which can actually be considered an informative indication because it may lead to a beneficial segmentation strategy.

### 3.2. Latent Class Analysis (LCA) Estimates and Profiles

We further analyzed the DCE choice data applying latent class analysis (LCA) to identify country-specific consumer segments with different product and process characteristics change preferences, and to compute parameter estimates quantifying each segment’s preference for the levels of each attribute. In contrast to the RPL models, LCA assumes unobserved preference heterogeneity after a discrete distribution, identifying for a distinct number of underlying classes of participants with similar preferences. In the present study, the Akaike information criterion (AIC), Bayesian information criterion (BIC), Chi-square, and log-likelihood measures were used to select the number of classes in this LCA (Table 5).

However, according to Boxall and Adamowicz [62] and Hole [72], there are no established absolute statistical solutions to select an optimal number of latent classes. Based on the interpretability and comparability of the DCE outputs between Hungary and Italy, the three latent classes solution in the present cross-country study was identified. The results of the three-class LCA models in both countries are reported in Table 6, based on the three attributes: Organic labels, Nutrition and health claims, and Price. Moreover, Table A2 in the Appendix A presents the results of the Kruskal–Wallis tests for country-specific consumer groups.

For all classes of the Hungarian and Italian sample, as expected, the coefficients for the price attribute were negative and statistically significant, suggesting that higher egg prices generate disutility. Participants in both countries prefer to purchase eggs carrying labels with the combination of the EU organic label and the national ones, with full nutrition and health claim are more likely to be in Class 1, namely Healthy Conscious Buyers. Participants from the segment of Healthy Conscious Buyers represent 46.4% of the Hungarian sample and 49.0% of the Italian sample. The coefficients of the constant in this consumer segment are strongly negative and significant in both countries, suggesting dislike for the opt-out alternatives shown in the DCE.

Participants in Class 2 in both countries, namely Price Sensitive and Quality Optimizing Opportunist Consumers, care mostly for the price and concern little for the information of nutrition and health claims, and rarely pay attention to organic labelling. Particularly, participants from Price Sensitive Consumers in Italy even exhibit insignificant part-worth utility for organic labelling. The relative importance of the nutrition and health claim is also quite low, although statistically significant, indicating that for these consumers, price represents really the main factor influencing choices.

The consumer segment of Class 3 in Hungary groups individuals who prefer not having the organic logo. Indeed, they show a statistically significant positive preference for eggs that carry no organic certification and a statistically significant negative preference for eggs labelled by the EU organic logo. Therefore, we named this segment Unlabelled Eggs Seekers. Regardless the presence of the nutrition and health claims, these consumers simply consider (low) prices for their choices, particularly if not associated with the EU organic logo label. Differently to Hungarian Class 3, participants from the Class 3 in Italy are in favor of buying EU organic certified eggs with no information regarding nutrition and health benefits. We named this segment Clean Labelling Consumers, since these individuals significantly dislike the labels with more (nutrition and health) information, preferring eventually the product with a simple EU organic logo. More importantly, the coefficients of the constant in Class 3 in both countries are positive and statistically significant, indicating that consumers prefer to select the “opt-out” alternative in the DCE.

According to the results of Kruskal–Wallis test presented in Table A2 in the Appendix A, we are able to further explore whether the three consumer segmentations significantly differ with respect to socio-demographics, behavior, and food value among the groups in the country-specific data. It is found that in Hungary, Health Conscious Buyers consist of participants who are mostly living in the urban area and in general purchase eggs infrequently compared to the other groups. For participants belong to the group of Unlabelled Eggs Seekers the share of people mainly dwelling in small size town, and rarely purchasing eggs with high in omega 3 fatty acids is significantly higher. Particularly, participants from this small segment significantly depreciate environmental-friendly and animal-friendly eggs (e.g., not paying attention to organic labels), and those high in omega 3 fatty acids. This can be explained that the Unlabelled Eggs Seekers might fulfil their needs for eggs by purchasing eggs unpacked, e.g., like those unlabelled purchased directly in rural areas from small scale producers (with up to maximum 50 laying hens in the farm). Moreover, the Price Sensitive and Quality Optimizing Opportunist Consumers involve participants who mainly live in large cities, buy organic eggs frequently, often purchase eggs with high in omega 3 fatty acids, and especially prefer to consume foods produced in an environmental friendly and animal-friendly manner.

The results of Kruskal–Wallis test in Table A2 in the Appendix A also show that in Italy Healthy Conscious Buyers purchase more often eggs with high in omega 3 fatty acids than the two other groups. Moreover, for these consumers it is important that the food eaten on a typical day is familiar, animal friendly, and a way of managing their mood (e.g., a good feeling or coping with stress) than the other classes. Price Sensitive Consumers buy less often organic eggs, and eggs at a farmers market or farm shop. For them, it is more important that the food eaten on a typical day is affordable, whilst it is less important that this food is environmentally friendly and produced and traded in a fair manner. Finally, Clean Labelling Consumers, buy more often eggs at a farmers market or farm shop, and find less relevant that the food eaten is a way of managing their mood.

### 3.3. Willingness to Pay Estimates

The results of country-specific segment-specific Willingness-to-Pay (WTP) estimates are presented in Table 7. In Hungary, Health Conscious Buyers are willing to pay a price premium of EUR 1.47 per 6-pack of eggs for the combination of the EU and national organic labelled eggs, and a WTP of EUR 1.63 for eggs with both nutrition and health claims, respectively. In addition, compared to the other groups the Price Sensitive and Quality Optimizing Opportunist Consumers demonstrate positive and relatively minor WTP for eggs carry either single EU organic label (EUR 0.27 per 6-pack of eggs) or a combination of the EU and national organic label (EUR 0.23 per 6-pack of eggs), as well as for eggs with either simplified nutrition claim (EUR 0.15 per 6-pack of eggs) or the full nutrition and health claim (EUR 0.51 per 6-pack of eggs). However, they would only buy eggs sold with no organic label (EUR −0.50) and no nutrition and health claims (EUR −0.66) with discounts. More interestingly, we also found that the Unlabelled Eggs Seekers are willing to pay a price premium (EUR 1.10 per 6-pack of eggs) for eggs with no label of production, which can be considered a significant premium as 80% of them pays less than EUR 2.0, while for these consumers to have the EU organic label on the package is disincentive as they would pay less for such eggs.

In Italy, the three classes presented a negative WTP for the product with no organic label, whilst only the Clean Labelling Consumers express a positive WTP (EUR 2.20 per 6-pack of eggs) for the EU organic label alone. This premium is higher than the price usually paid by half of the respondents in this group, i.e., EUR 1.70 per 6-pack of eggs. The Healthy Conscious Buyers report a positive and significant WTP of EUR 2.52 per 6-pack of eggs for the combination of EU organic and national (private certification body) label. This premium is relatively high considering that 55% of the respondents in this group usually pay an average price lower than EUR 2.00 per 6-pack of eggs. Considering the nutrition and health claim, the Healthy Conscious Buyers and the Price Sensitive Consumers report a negative WTP for the product with no label, indicating a preference for having at least the nutrition claim displayed on the package. In particular, Healthy Conscious Buyers expressed a high premium for the full nutrition and health claim (EUR 4.07 per 6-pack of eggs), especially if compared to the price normally paid by these consumers. The Price Sensitive Consumers report a moderately positive and significant WTP for the product with the nutrition and health claim (EUR 0.51). On the other hand, the Clean Labelling Consumers indicate a positive and significant WTP only for the product without any nutrition and health claim, showing a disutility for providing this information on the package.

## 4. Discussion

The present study utilized DCEs to assess Hungarian and Italian consumers’ interest in and WTP for organic label and nutrition and health claims considering a standard 6-pack of eggs as a specific food object. This paper contributed significantly to the understanding of the synergies between organic food labels with nutrition and health claims, and of the heterogeneous consumers’ preferences. Using an LCA modelling approach, we have identified three distinct segments among respondents in each country, revealing heterogeneous preferences and WTP for organic labels and nutrition and health claims, as well as different socio-demographic and behavioral characteristics.

The results in both countries show a general preference of consumers for the EU organic logo combined with the national one, as compared to the EU organic logo alone or to the product with no label. Similarly, in both countries, consumers prefer the full nutrition and health claim (i.e., High in Omega 3 fatty acids, contributes to the maintenance of normal function of the heart and normal blood pressure), compared to the nutrition information alone (i.e., High of Omega 3 fatty acids) or to the product with no additional information. However, we should consider that more educated and urban consumers, like those included in the samples, might understand and use nutrition and health claims, as well as organic labels, more than less educated and rural ones [73,74].

However, a more detailed LCA indicates significant differences across segments of respondents. In particular, in Hungary and Italy, respectively, 46.4% and 49.0% of the sample has been grouped in the Healthy Conscious Buyers with high and positive WTP estimates for the EU organic and national organic labels as well as the nutrition and health claims. In Italy, in particular, this segment presents a stronger familiarity with the organic labelled products and with foods carry nutrition claims. Other studies have documented that familiarity affects both organic consumption and WTP for organic food [30]. In other words, those regularly purchasing organic products and foods with nutrition claims are also willing to spend more on these products. The EU organic logo is generally more familiar in the Italian market, where no other governmental nor a dominant private organic logo existed. It was suggested that this factor could also explain the positive attitude and trust of Italian consumers towards the EU logo [32], in particular if associated with the certification body one. Possibly the non-significant effect of this attribute for 51% of the sample, represented in the Price Sensitive and Clean Labelling segments, could be due to the absence of dominant organic labels of private companies in the Italian organic market.

Therefore, synergies between organic and nutrition labels have emerged in both countries, at least in the Healthy Conscious Buyers segment. In Hungary also the Price Sensitive and Quality Optimizing Opportunist Consumers (46% of the sample) exhibit positive and significant effects for both labels. In other studies, organic consumers have shown a higher level of sustainability concern in their general food choices, including principles related to a more sustainable lifestyle, as well as health aspects and naturalness of food [29,34]. Similarly, consumers often perceive organic eggs healthier than conventional products, and they are willing to pay more for them [13,35,36,37]. Synergies between sustainability and health-related labels have also been reported in Italy and other European countries for several fish species, including seabream, seabass and herrings [75].

On the other hand, consumers grouped in the Unlabelled Eggs Seekers segment in Hungary and in the Clean Labelling Consumers segment in Italy reported non-significant or negative effects. This finding is in line with the study reported by [14] that consumers do not appreciate the value added of organic eggs, presenting a decreasing marginal utility for added attributes. As suggested in other cases (see, e.g., Grunert, et al. [76]), low values of WTP for eco-friendly and healthy-related attributes could be due to consumers’ perception of the effectiveness of these attributes, and not by the low interest or value per se. In Hungary, the unfavorable attitude towards the EU organic logo and nutrition claims of the Unlabelled Eggs Seekers segment can be also explained by their shopping behavior of rarely purchasing eggs that are organic certified and high in omega 3 fatty acid, and by their need for unlabelled eggs to be available in Hungarian farmers’ market and at small scale producers in short food supply chains. Thus, labels (both trademarks and quality marks) only have a very limited impact on consumers’ attitude as motivators to purchase organic food [24] in Hungary.

Gender does not represent a factor significantly affecting the probability of belonging to a specific segment in both countries, the same finding holds for both age and educational level. Particularly, in Hungary rather few socio-demographics are able to explain the probability of belonging to one of the three segments. Although the Unlabelled Eggs Seekers show considerably low interest of buying eggs certified organically, with additional nutritional value (e.g., omega 3 fatty acid) and produced environmental-friendly and animal-friendly, there is still unclear evidence whether the groups between Healthy Conscious Buyers and Price Sensitive and Quality Optimizing Opportunist Consumers in Hungary possess distinguishable attitudes towards organic labelled eggs or eggs with nutrition claims. This partially contradicts the results of previous studies indicating that a typical Hungarian organic food consumer is female, belongs to the upper-income level segment, and is committed to a healthy lifestyle and environmental protection [22,24]. In Italy, although few socio-demographics are significantly different across the three segments, a different attitude was reported. Healthy Conscious Buyers, for instance, are more interested that the food eaten on a typical day is familiar, animal friendly, and able of managing their mood than the other classes, whilst Price Sensitive Consumers prefer that the food eaten on a typical day is affordable. This latter segment is less interested that this food is environmentally friendly and produced and traded in a fair manner. These two segments, representing almost 90% of the sample, identify two different approaches to food choices. On the one hand, a segment of consumers more involved in the nutritional and ethical dimension of food purchase decisions, and on the other hand, a segment more interested in the convenience and economic effects of food choices. These behaviors are not uncommon and have already been reported in other studies showing that Italian organic consumers are more concerned with the sustainability dimensions of their general food choices, and have a more sustainable lifestyle [29,34].

## 5. Conclusions

The study presented is insightful in the scope of consumer behavior research as well as in practice for producers, traders and other stakeholders in the food market; there are several implications of these findings. Sustainable food choices must be affordable and able to meet the need of different segments of society. In Italy and Hungary, approx. 40–45% of the sample is more interested in the affordability of food choices than in their environmental and health consequences. The sustainability of dietary choices must necessarily face all the environmental, social and economic aspects [77]. It is not uncommon that organic products are perceived as being more expansive than conventional ones, and this is the most significant barrier why they are not consumed widely [20,21]. Moreover, the fact that the organic certified labels seem to be less important should be considered seriously, as this might indicate that there is a lack of trust in the organic certification system in general and/or a label of knowledge with respect to what organic implies. Subsidies or voucher programs should encourage healthy and sustainable dietary choices, particularly to enable their adoption in low-income and middle-income segments of the population [78,79]. For companies, the synergies found in the Healthy Conscious Buyers segments for eco-friendly and healthy-related choices suggest the possibility of combining these labels to meet the expectations of consumers more involved in the ethical and nutritional dimensions of purchase decisions [75]. As it is found that not all (nutrition and health) claims are similarly appealing to consumers from different countries such as in Hungary and Italy, food producers and processors should consider tailoring food claims as well as food labelling of their products to country-specific demands, refining in that way the effectiveness of claim-based/label-based marketing communications. Finally, the identified segments of the Healthy Conscious Buyers, Price Sensitive and Quality Optimizing Opportunist Consumers, the Clean Labelling Consumers, and the Unlabelled Eggs Seekers represent a structured profile of the European (egg) shoppers, suggesting the proportions of consumers holding similar consumption patterns of preferences around which marketing strategy could be designed that would further facilitate communication development towards sustainable and healthier food choices. This might suggest that companies should implement strategies combining these elements (e.g., in communication campaigns) for selling their product in premium segments of the market.

Some limitations of the study should be also highlighted. The use of hypothetical choice experiments may have inflated the WTP estimates, and future analysis could benefit from triangulation with revealed preference data obtained, for instance, in real settings (e.g., auction-based experiments). On the other hand, this allowed us to obtain a large amount of data; moreover, the use of cheap talk scripts should have minimized any hypothetical bias. Second, we focused only on three attributes, ignoring other elements usually used by consumers in their food choices, e.g., brands, other production practices (e.g., free-range breeding). Therefore, the results of this research do not claim to cover the whole complex eggs market, whereas they provide practitioners and actors in the egg industry with more insights to understand consumer motivations when buying eggs carrying less investigated features, such as sustainability and health-related attributes. Third, in the present study, we only focus on one product, eggs. Thus, our outputs hold only for this animal product. For a better understanding of the relevance of organic labels and nutrition claims on consumers’ purchase decision of across other product categories, additional investigations are needed in the future research. Finally, the authors suggest that for future research, it is worth addressing the attribute non-attendance (ANA) incidence, as the ANA can occur when a respondent simplifies the choice task by ignoring an attribute [80], and this simplification could become an issue, because ignoring an attribute may alter the effect of the DCE. Confidently, our theoretical and experimental approach can be indeed applied to other product categories in the future.

## Figures and Tables

**Table 1:** Attributes and respective levels used in the choice experiment.

Country	Attributes and Respective Levels
**Hungary**	**Organic labels:** No-label EU organic label 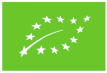 EU organic label and Hungarian organic label 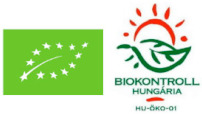 **Nutrition and health claims:** No claim High of Omega 3 fatty acids (nutrition claim) High of Omega 3 fatty acids, contributes to maintenance of normal function of the heart and normal blood pressure (nutrition and health claim) **Price (HUF/6 eggs box):** 189 HUF (c.a. EUR 0.62) 279 HUF (c.a. EUR 0.91) 369 HUF (c.a. EUR 1.19) 459 HUF (c.a. EUR 1.48)
**Italy**	**Organic labels:** No-label EU organic label 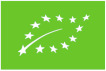 EU organic label and Hungarian organic label 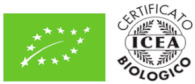 **Nutrition and health claims:** No claim High of Omega 3 fatty acids (nutrition claim) High of Omega 3 fatty acids, contributes to maintenance of normal function of the heart and normal blood pressure (nutrition and health claim) **Price (HUF/6 eggs box):** EUR 1.50 EUR 1.90 EUR 2.30 EUR 2.70

**Table 2:** Sample structure across the three countries: Hungary and Italy.

	Hungary		Italy		All
	Sample	Nat. Avg. ^a^	Sample	Nat. Avg.	
Valid *N*	403		404		807
Gender					
Female (%)	49.6	52.5	52.2	51.3	50.9
Male (%)	50.4	47.5	47.8	48.7	49.1
Average age	42.2	41.4	43.2	44.9	42.7
Living area					
Rural area (%)	15.4	30.5	13.9	24.3	14.6
Urban medium town (%)	37.5	34.4	45.3	42.4	41.4
City (%)	47.1	35.1	40.8	33.3	44.0
Education					
Lower secondary/primary education or below (%)	1.7	4.9	6.9	39.9	4.3
Upper secondary education (%)	31.3	48.1	39.9	41.5	35.6
University or college entrance qualification (%)	24.6	30.1	17.3	0.9	20.9
Bachelor’s degree or equivalent level (%)	26.1	10.1	13.4	3.6	19.7
Master, Postgraduate or doctoral degree (%)	16.4	6.8	22.5	14.1	19.5
Household monthly net income		222,000 HUF		EUR 2616	
(HU) < 150,000 HUF (c.a. EUR 486)/(IT) < EUR 900 (%)	10.2		5.9		8.1
(HU) 150,001–205,000 HUF (c.a. EUR 487–EUR 664) /(IT) EUR 900–EUR 1,500 (%)	14.9		20.8		17.8
(HU) 205,001–235,000 HUF (c.a. EUR 665–EUR 761) /(IT) EUR 1501–EUR 2500 (%)	9.4		28.7		19.1
(HU) 235,001–380,000 HUF (c.a. EUR 762–EUR 1231) /(IT) EUR 2501–EUR 3500 (%)	31.0		18.3		24.7
(HU) 380,001–835,000 HUF (EUR 1232–EUR 2705) /(IT) EUR 3501–EUR 4500 (%)	25.3		9.2		17.2
(HU) ≥ 835,001 HUF (c.a. EUR 2706)/(IT) ≥ EUR 4501 (%)	2.7		3.2		3.0
Prefer not to answer (%)	6.5		13.9		10.2
Household size	2.96	2.86	2.94	2.40	2.95
Number of children (<18 year) in a household	0.59	1.06	0.54	0.50	0.56

^a^ Nat. Avg. = National average in Hungary and in Italy, respectively.

**Table 3:** Attribute importance scores for Hungary and Italy.

Country	Hungary	Italy
*N*	403	404
	Avg. Imprt. (S.D.)	Avg. Imprt. (S.D.)
Organic labels	17.34 (12.23)	17.17 (12.63)
Nutrition and health claims	23.87 (15.70)	29.82 (17.00)
Price	58.79 (21.61)	53.01 (22.78)

Avg. Imprt. = Average Importance, S.D. = Standard Deviation.

**Table 4:** Empirical results from random parameter logit models of the cross-country DCE data.

Country	Hungary	Italy
*N*	403	404
Null Log-likelihood	−3352.05	−3360.37
Restricted Log-likelihood	−2538.72	−2447.81
AIC	5091.45	4909.63
BIC	5119.44	4937.62
McFadden Pseudo R^2^	0.24	0.27
	Avg. Utilities (S.D.)	Avg. Utilities (S.D.)
Organic labels		
No label	−14.48 (32.54 ***)	−21.54 (23.58 ***)
EU organic	0.34 (19.47 ***)	2.53 (14.49 **)
EU organic + national organic	14.13 (20.38 ***)	19.01 (24.49 ***)
Nutrition and health claims		
No-claim	−24.04 (42.32 ***)	−32.36 (44.58 ***)
Nutrition claim	10.05 (21.78 ***)	5.30 (23.01 ***)
Nutrition and health claim	13.99 (30.52 ***)	27.06 (37.24 ***)
Price		
	−54.12 (31.56 ***)	−47.20 (33.21 ***)
Constant	−190.80 (194.06 ***)	−160.75 (181.33 ***)

**,***; *p*-value of the random parameter’s standard deviation < 0.01, 0.001; S.D. = Standard Deviation. Note 1: The reported average utilities are zero-centred; Note 2: The WTP measure derived from the RPL models can be found in Appendix A,Table A1.

**Table 5:** Summary of measures for selecting the optimal number of classes.

	Number of Groups	Log-Likelihood	AIC	BIC	Chi-Square
Hungary (*N* = 403)	2	−2203.38	4432.75	4508.03	2297.37
	3	−2037.72	4115.44	4231.26	2628.67
	4	−1954.10	3962.20	4118.54	2795.92
	5	−1930.24	3928.49	4125.37	2843.63
Italy (*N* = 440)	2	−2280.17	4586.34	4661.65	2160.41
	3	−2097.40	4234.80	4350.66	2525.96
	4	−2026.22	4106.43	4262.85	2668.32
	5	−1989.34	4046.68	4243.65	2742.07

**Table 6:** Latent class model.

	Hungary						Italy					
*N*	403						404					
Null log-likelihood	−3352.05						−3360.37					
Restricted log-likelihood	−2037.72						−2097.39					
AIC	4115.44						4234.80					
BIC	4231.26						4350.66					
Chi-Square	2628.67						2525.95					
	Group 1 Health Conscious Buyers		Group 2 Price Sensitive and Quality Optimizing Opportunist Consumers		Group 3 Unlabelled Eggs Seekers		Group 1 Health Conscious Buyers		Group 2 Price Sensitive Consumers		Group 3 Clean Labelling Consumers	
Group size	46.4%		45.6%		8.0%		49.0%		39.5%		11.6%	
	Relat. Imprt. (%)	Part-worth utilities (t-stat.)	Relat. Imprt. (%)	Part-worth utilities (t-stat.)	Relat. Imprt. (%)	Part-worth utilities (t-stat.)	Relat. Imprt. (%)	Part-worth utilities (t-stat.)	Relat. Imprt. (%)	Part-worth utilities (t-stat.)	Relat. Imprt. (%)	Part-worth utilities (t-stat.)
Organic labels	20.30		9.70		21.85		23.86		4.59		19.82	
No label		−25.48 *** (−3.08)		−18.82 *** (−7.02)		35.49 *** (2.99)		−33.51 *** (−6.90)		−8.78 *** (−3.11)		−33.26 *** (−2.60)
EU organic		−9.95 (−1.09)		10.28 *** (3.49)		−30.07 * (−2.03)		−4.58 (0.88)		5.00 (1.55)		26.20 * (2.18)
EU organic and national organic		35.43 *** (4.50)		8.54 *** (3.32)		−5.42 (−0.40)		38.09 *** (8.95)		3.79 (1.32)		7.06 (0.56)
Nutrition and health claims	31.40		14.69		13.88		45.91		16.08		56.32	
No-claim		−54.78 *** (−6.30)		−24.85 *** (7.90)		15.24 (1.23)		−76.23 *** (−13.32)		−27.99 *** (−8.09)		110.70 *** (9.72)
Nutrition claim		15.37 (1.78)		5.64 * (1.98)		11.14 (0.87)		14.74 *** (2.98)		7.72 ** (2.43)		−58.26 *** (−3.75)
Nutrition and health claim		39.41 *** (5.11)		19.21 *** (7.01)		−26.39 (−1.89)		61.49 *** (14.75)		20.27 *** (6.69)		−52.44 *** (−3.39)
Price	48.30	−48.30 *** (-9.60)	75.61	−75.61 *** (18.41)	64.27	−64.27 *** (5.89)	30.23	−30.23 *** (−10.53)	79.32	−79.32 *** (−18.53)	23.86	−23.86 *** (−3.16)
Constant		−475.76 *** (−12.63)		−39.90 *** (4.87)		161.25 *** (0.97)		−264.96 *** (−11.46)		−11.32 (−1.82)		90.35 *** (6.61)

Relat. Imprt. = Relative importance; *, **, ***; *p* < 0.05, 0.01, 0.001.

**Table 7:** Mean WTP estimates for cross-country latent consumer segments.

Country	Hungary			Italy		
*N*	403			404		
Segment label	Group 1 Health Conscious Buyers	Group 2 Price Sensitive and Quality Optimizing Opportunist Consumers	Group 3 Unlabelled Eggs Seekers	Group 1 Health Conscious Buyers	Group 2 Price Sensitive Consumers	Group 3 Clean Labelling Consumers
Segment size	46.40%	45.60%	8.00%	49.00%	39.50%	11.60%
	WTP	WTP	WTP	WTP	WTP	WTP
Organic labels						
No label	−1.06 ***	−0.50 ***	1.10 ***	−2.22 ***	−0.22 ***	−2.79 ***
EU organic	−0.41	0.27 ***	−0.94 *	−0.30	0.13	2.20 *
EU organic and national organic	1.47 ***	0.23 ***	−0.17	2.52 ***	0.10	0.59
Nutrition and health claims						
No-claim	−2.27 ***	−0.66 ***	0.47	−5.04 ***	−0.71 ***	9.28 ***
Nutrition claim	0.64	0.15 *	0.35	0.98 ***	0.19 **	−4.88 ***
Nutrition and health claim	1.63 ***	0.51 ***	−0.82	4.07 ***	0.51 ***	−4.40 ***

Relat. Imprt. = Relative importance; *, **, ***; *p* < 0.05, 0.01, 0.001.

## Appendix A

**Table A1:** WTPs derived based on the RPL models.

Country	Hungary	Italy
*N*	403	404
	WTP	WTP
Organic labels		
No label	−0.54	−0.91
EU organic	0.01	0.11
EU organic and national organic	0.52	0.81
Nutrition and health claims		
No-claim	−0.89	−1.37
Nutrition claim	0.37	0.22
Nutrition and health claim	0.52	1.15

**Table A2:** Cross-country consumer segment profiles and the results of the Kruskal–Wallis test.

N	Hungary				Italy			
	403				404			
	Group 1 *Health Conscious Buyers*	Group 2 *Price Sensitive and Quality Optimizing Opportunist Consumers*	Group 3 *Unlabelled Eggs Seekers*		Group 1 *Health Conscious Buyers*	Group 2 *Price Sensitive Consumers*	Group 3 *Clean Labelling Consumers*	
	46.40%	45.60%	8.00%	K-W test	49.00%	39.50%	11.60%	K-W test
Gender								
Female (%)	46.6	49.6	50		51.0	53.7	52.2	
Male (%)	53.4	50.4	50		49.0	46.3	47.8	
Age	42.2	42.2	45.6		43.5	42.4	44.4	
Living location				*				
Rural area (%)	13.6	15.4	25.0		10.8	13.4	28.3	
Small size town (%)	42.4	37.5	46.9		46.4	46.3	37.0	
Large city (%)	44.0	47.1	28.1		42.8	40.2	34.8	
Education								
Up to secondary school education (%)	36.1	25.0	31.1		52.2	48.2	44.3	
University degree, vocational training or higher (%)	63.9	75.0	68.9		47.8	51.8	55.7	
How often do you buy organic egg? (1 indicates Never, 7 indicates Every time)	2.7	3.1	2.6	*	4.6	3.8	4.5	***
How often do you buy eggs with high in omega 3 fatty acids? (1 indicates Never, 7 indicates Every time)	2.6	3.0	1.8	***	3.4	2.6	2.3	***
How often do you buy eggs at a farmers market or farm shop? (1 indicates Never, 7 indicates Every time)	4.4	4.6	4.6		3.9	3.4	4.2	**
What price do you normally pay for a 6-pack of eggs?				***				
(HU) Below c.a. EUR 0.49/(IT) Below 1.40 EUR (%)	0.5	1.7	9.4		3.6	23.2	13.0	
(HU) Between c.a. EUR 0.50–EUR 0.72/(IT) Between 1.40 EUR and 1.70 EUR (%)	36.1	19.4	34.4		21.1	42.1	37.0	
(HU) Between c.a. EUR 0.72–EUR 0.94/(IT) Between 1.71 EUR and 2.00 EUR (%)	40.3	33.3	25.0		29.4	22.0	17.4	
(HU) Between c.a. EUR 0.95–EUR 1.17/(IT) Between 2.01 EUR and 2.30 EUR (%)	9.9	22.8	9.4		19.6	3.7	4.3	
(HU) Between c.a. EUR 1.18–EUR 1.40 (IT) Between 2.31 EUR and 2.60 EUR (%)	9.4	11.1	6.3		10.3	3.0	15.2	
(HU) Between c.a. EUR 1.41–EUR 1.62/(IT) Between 2.61 EUR and 2.90 EUR (%)	1.0	8.3	3.1		5.2	0.0	0.0	
(HU) More than c.a. EUR 1.63/(IT) More than 2.90 EUR (%)	1.0	2.2	3.1		2.1	0.0	2.2	
Do not remember (%)	1.6	1.1	9.4		8.8	6.1	10.9	
How often do you buy eggs?				**				
About or Less than three times a month (%)	72.8	56.7	59.4		63.4	61.6	65.2	
About or more than once a week (%)	27.2	43.3	40.6		36.6	38.4	34.8	
How often do you eat eggs?								
Once a week or les (%)	45.0	45	50.0		63.4	69.5	71.7	
More than once a week (%)	55.0	55.0	50.0		36.6	30.5	28.3	
It is important to me that the food I eat on a typical day… (1 indicates strongly disagree and 7 strongly agree)								
…is healthy.	5.65	5.73	5.06		5.79	5.71	5.57	
…is a way of managing my mood (e.g., a good feeling or coping with stress).	4.95	5.07	4.47		5.18	4.92	4.52	***
…is convenient (in buying and cooking).	5.34	5.28	4.84		5.19	5.20	4.91	
…provides me with pleasure (e.g., appearance, texture, smell, taste).	5.67	5.57	5.13		5.74	5.77	5.41	
…is natural (no additives, only natural ingredients).	5.35	5.56	5.19		5.63	5.41	5.50	
…is affordable.	6.15	5.84	5.69		4.54	5.15	4.76	***
…helps me control my weight.	4.74	4.87	4.44		5.14	4.95	4.83	
…is familiar.	5.16	5.16	4.75		4.94	4.60	4.35	**
…is environmentally friendly.	5.09	5.41	4.81	*	5.36	5.00	5.24	**
…is animal friendly.	4.68	5.23	4.38	***	5.51	5.15	5.28	**
…is produced and traded in a fair manner.	5.07	5.27	5.88		5.18	4.74	5.20	***
Monthly net household income								
(HU) < 150,000 HUK (c.a. EUR 486)/(IT) < EUR 900 (%)	11.5	3.1	3.1		3.6	8.5	6.5	
(HU) 150,001–205,000 HUF (c.a. EUR 487–EUR 664)/(IT) EUR 900–EUR 1500 (%)	14.1	18.8	18.8		19.6	22.6	19.6	
(HU) 205,001–235,000 HUF (c.a. EUR 665–EUR 761)/(IT) EUR 1501–EUR 2500 (%)	11.0	6.3	6.3		26.3	31.1	30.4	
(HU) 235,001–380,000 HUF (c.a. EUR 762–EUR 1231)/(IT) EUR 2501–EUR 3500 (%)	29.8	28.1	28.1		22.2	14.6	15.2	
(HU) 380,001–835,000 HUF (EUR 1232–EUR 2705)/(IT) EUR 3501–EUR 4500 (%)	25.7	28.1	28.1		12.4	5.5	8.7	
(HU) ≥ 835,001 HUF (c.a. EUR 2706)/(IT) ≥ EUR 4501 (%)	2.1	6.3	6.3		3.6	3.7	0.0	
Prefer not to answer (%)	5.8	9.4	9.4		12.4	14.0	19.6	
Household size	2.96	2.93	3.09		2.93	2.95	2.89	
Number of kids	0.61	0.56	0.63		0.57	0.47	0.65	

***, **, * Demographics are significant at 0.1%, 1%, 5%, level with asymptotic method of non-parametric Kruskal–Wallis test among cross-country three latent segment.
